# Comparative Evaluation of Six Commercialized Multiplex PCR Kits for the Diagnosis of Respiratory Infections

**DOI:** 10.1371/journal.pone.0072174

**Published:** 2013-08-23

**Authors:** Sylvie Pillet, Marina Lardeux, Julia Dina, Florence Grattard, Paul Verhoeven, Jérôme Le Goff, Astrid Vabret, Bruno Pozzetto

**Affiliations:** 1 Laboratory of Bacteriology and Virology, University Hospital of Saint-Etienne, Saint-Etienne, France; 2 Laboratory of Virology, University Hospital of Caen, Caen, France; 3 Université Paris Diderot, Sorbonne Paris Cité, Microbiology Department, Saint-Louis Hospital, APHP, Paris, France; University of Hong Kong, Hong Kong

## Abstract

The molecular diagnosis of respiratory infection can be performed using different commercial multiplex-based PCR kits whose performances have been previously compared individually to those of conventional techniques. This study compared the practicability and the diagnostic performances of six CE-marked kits available in 2011 on the French market, including 2 detecting viruses and atypical bacteria (from Pathofinder and Seegene companies) and 4 detecting only viruses (from Abbott, Genomica, Qiagen and Seegene companies). The respective sensitivity, specificity, accuracy and agreement of each multiplex technique were calculated by comparison to commercial duplex PCR tests (Argene/bioMérieux) used as gold standard. Eighty-eight respiratory specimens with no pathogen (n = 11), single infections (n = 33) or co-infections (n = 44) were selected to cover 9 viruses or groups of viruses and 3 atypical bacteria. All samples were extracted using the NUCLISENS® easyMAG™ instrument (bioMérieux). The overall sensitivity ranged from 56.25% to 91.67% for viruses and was below 50% with both tests for bacteria. The overall specificity was excellent (>94% for all pathogens). For each tested kit, the overall agreement with the reference test was strong for viruses (kappa test >0.60) and moderate for bacteria. After the extraction step, the hands-on time varied from 50 min to 2h30 and the complete results were available in 2h30 to 9 h. The spectrum of tested agents and the technology used to reveal the PCR products as well as the laboratory organization are determinant for the selection of a kit.

## Introduction

The global burden of acute respiratory infection (ARI) remains a huge problem of Public Health. In developed countries, the number of viral respiratory episodes per year has been estimated between 6 and 10 in children before school age versus 3 to 5 in those after this age and ARI represents the cause of 30 to 40% of hospital admissions in this category of patients [1] [2]. A wide range of pathogens are involved in ARI, including bacteria and viruses.

The diagnosis of ARI relies both on clinical examination, radiological exploration and biological nonspecific inflammatory tests (including the level of protein C reactive or procalcitonin). The identification of the causative agent(s) is often omitted or limited to a few pathogens easy to detect by rapid antigen direct tests (influenza viruses and respiratory syncytial virus (RSV) in respiratory specimens, *Streptococcus pneumoniae* and *Legionella pneumophila* in urine specimens). However, the distinction between viral and bacterial infections is often impossible by using non-microbiological criteria [3]–[4].

The need for precise and rapid identification of the causative agents of ARI has been reviewed recently [2], [5]–[6]. The main advantages of this strategy are (i) a better use of antimicrobials including antiviral drugs and antibiotics [3], [7] and thus limiting the development of bacterial resistance, (ii) the reduction of unnecessary paraclinical explorations and of the duration of hospitalization [8], (iii) the rapid implementation of isolation measures when necessary, thus limiting the risk of nosocomial transmission, (iv) the collection in real time of new epidemiological data on the seasonal spread of pathogens, and (v) the identification of simultaneous or successive infections [6], [9]–[10] that may justify specific intervention or explain the severity of the clinical picture.

The detection of agent’s genome responsible for respiratory infection have been revolutionized by recent advances in the field of nucleic acid amplification tests and notably of multiplex PCR [4], [11]–[14]. In addition to their excellent sensitivity, much superior to that of conventional techniques [15], these techniques allow the simultaneous detection of a wide range of pathogens, mostly viruses [16]–[19], but also atypical bacteria (including *Mycoplasma pneumoniae*, *Chlamydophila pneumoniae*, *L. pneumophila* and *Bordetella pertussis*) [20]–[23], with a short-time return of the results to clinicians.

The objective of the present study was to evaluate the technical performances of six commercial kits based on multiplex PCR and available on the European market at the beginning of 2011 for the diagnosis of respiratory infection. The kits were compared to a combination of biplex PCR tests used as gold standard on a panel of respiratory secretions selected for their content in various infectious agent(s). The performances of each kit for detecting different respiratory pathogens, including sensitivity, specificity, accuracy and agreement, were evaluated for each agent; other technical properties were also taken into consideration. Globally, the kits whose commercial development is still pursued were shown to be convenient for routine use in a clinical laboratory setting.

## Materials and Methods

### Presentation of the Kits used in this Study

**Figure 1:**
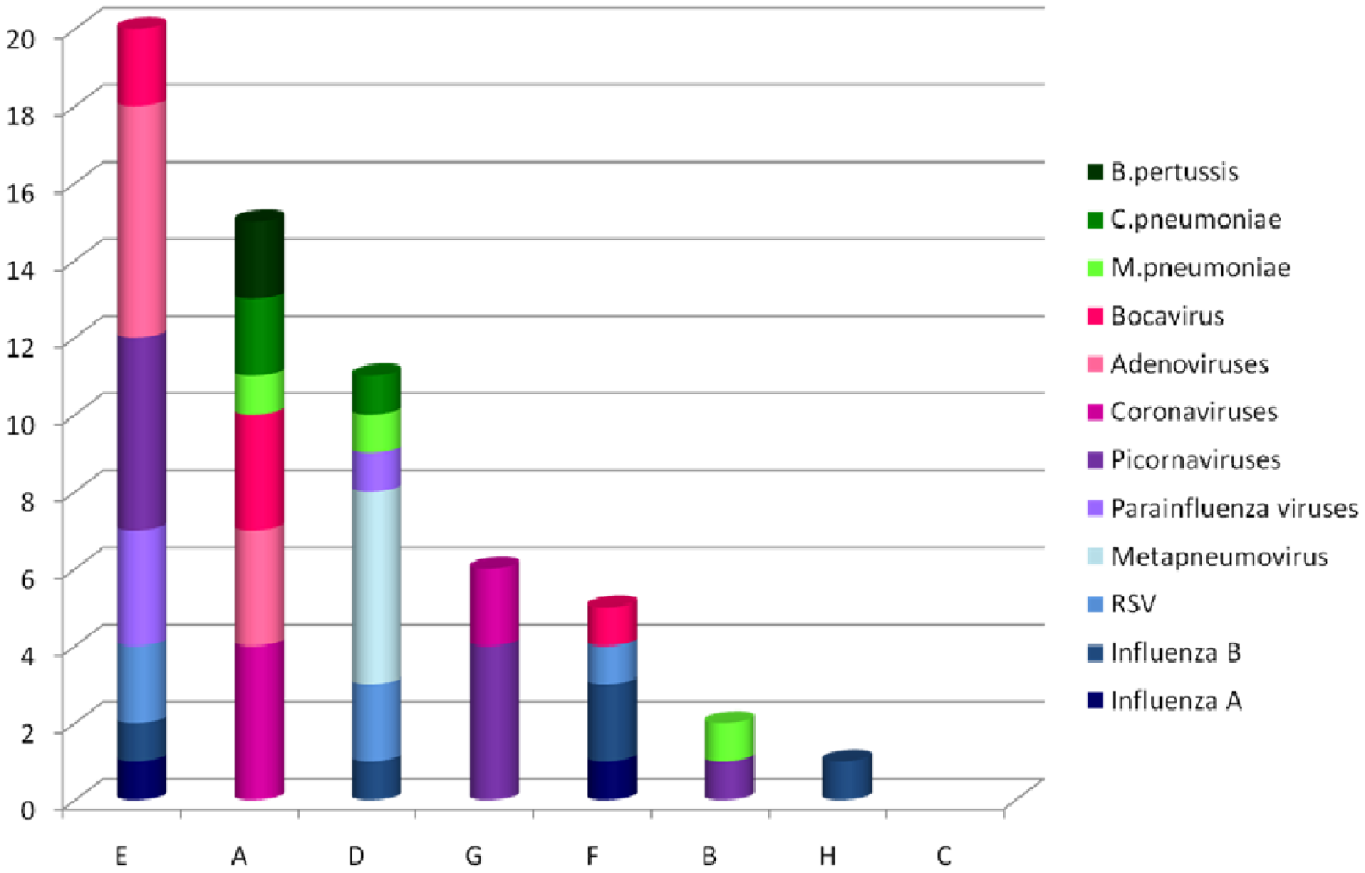
Schematic synopsis of the design of the study. (See text for correspondence between letters and commercial denominations).

Six kits based on multiplex detection of respiratory viruses or atypical bacteria and available in the French market in 2011 were tested. All were used as recommended by the manufacturers. The technologies are depicted in Figure 1. Letters A to F were used to design the kits in the following sections. Kit A corresponds to RespiFinder® SMART 22 (Pathofinder, Maastricht, The Netherlands); the reverse transcription and preamplification steps were performed on GeneAmp® PCR system 2700 (Applied Biosystems) and the hybridization, ligation and detection steps on the LightCycler®480 system (Roche Applied Science). Kit B corresponds to the Seeplex® RV15 OneStep ACE Detection and Pneumobacter ACE Detection (Seegene Inc., Seoul, South Korea); a GeneAmp® PCR system 9700 (Applied Biosystems) was used for amplification and the size of PCR products was read on a TapeStation after electrophoresis using ScreenTape. Kit C corresponds to the Magicplex RV Panel Real-time Test (Seegene Inc); the amplification was performed using GeneAmp® PCR system 9700 (Applied Biosystems) and the detection of PCR products was done on ABI7500 (Applied Biosystems). Kit D corresponds to Clart® Pneumovir (Genomica, Madrid, Spain); the amplification was performed on GeneAmp® PCR system 2700 (Applied Biosystems) and the hybridization of PCR products on the array was read using the Clinical Array System (CAR) (Genomica). Kit E corresponds to xTAG® respiratory Viral Panel fast (Abbott, Rungis, France) and kit F to ResPlex II Panel v2.0 (Qiagen, Hilden, Germany), for these two kits, the amplification was performed on GeneAmp® PCR system 9700 (Applied Biosystems) and the detection of PCR products on a Luminex platform (Bio-Plex, Bio-Rad).

The test used as gold standard for evaluating the six kits described above corresponds to a combination of 7 duplex Respiratory Multi Well System r-gene™ (Influenza A/B, RSV/hMPV, Rhino&EV/CC, AD/hBoV, Chla/Myco pneumo, HCoV/HPIV and *Bordetella*) commercialized by Argene/bioMérieux (Marcy l’Etoile, France). The real-time PCR reactions were performed on an ABI7500fast (Applied Biosystems).

The viruses and/or bacteria that could be detected by the 7 kits mentioned above are listed in Table 1.

**Table 1:** Presentation of the 7 kits used in the study for detecting viruses (see text for correspondence between letters and commercial denominations).

	Gold standard	A	B	C	D	E	F
Influenza A viruses	Yes	Yes w diff A(H1N1)pdm09	Yes	Yes	Yes w diff A(H1N1)pdm09	Yes w diff (H1 and H3)	Yes
Other influenza viruses	B	B	B	B	B and C	B	B
Respiratory syncytial virus	Yes	Yes w diff	Yes w diff	Yes	Yes w diff	Yes	Yes w diff
Metapneumovirus	Yes	Yes	Yes	Yes	Yes	Yes	Yes
Parainfluenza viruses	Yes	Yes w diff	Yes w diff	Yes w diff	Yes w diff	Yes w diff	Yes w diff
Picornaviruses (rhinovirus/enterovirus)	Yes	Yes	Yes w diff betweenrhinovirus A, B and C,and enterovirus	Yes	Yes w diff rhinovirus and enterovirus B	Yes	Yes w diff rhinovirus and coxsackie virus/echovirus
Coronaviruses	Yes	Yes w diff betweenthe 4 types	Yes (except HKU1) w diffbetween OC43and 229E/NL63	Yes (except HKU1) wdiff between the3 types	Detection of 229E only	Yes w diff betweenthe 4 types	Yes w diff between the 4 types
Adenoviruses	Yes (A to G)	Yes	Yes	Yes	Yes	Yes	Yes (B and E only)
Bocavirus	Yes	Yes	Yes	Yes	Yes	Yes	Yes
*Mycoplasma pneumoniae*	Yes	Yes	Yes	No	No	No	No
*Chlamydophila pneumoniae*	Yes	Yes	Yes	No	No	No	No
*Bordetella pertussis*	Yes	Yes	Yes	No	No	No	No

Yes: group of pathogens detected; w diff: with differentiation between types. No: group of pathogens not detected.

### Selection and Preparation of the Respiratory Samples

In order to constitute a representative panel of specimens containing a wide range of the respiratory pathogens (viruses and atypical bacteria) tested in the evaluated kits, 88 respiratory samples (30 from the University Hospital of Caen and 58 from the University Hospital of Saint-Etienne, France; one half being nasal swab and one half being respiratory secretions) were selected (by S.P., F.G., J.D. and A.V.) for the study. Viral and bacterial pathogens were routinely detected by immunofluorescence assay (respiratory syncytial viruses, influenza viruses, parainfluenza viruses, adenoviruses, and metapneumoviruses in Saint-Etienne) or molecular methods (home-brew method for influenza A H1N1pdm09 [24] in Saint-Etienne; Chlamylege kit from Argene/bioMérieux [20] for atypical bacteria in Saint-Etienne; home-brew molecular methods for viruses (rhinoviruses/enteroviruses, adenoviruses, bocavirus, coronaviruses, parainfluenza viruses and metapneumoviruses) in Caen [11], [15], [25]–[26]). The criteria of selection were (i) the presence of clinical ARI, (ii) the detection of a respiratory pathogen by the routine tests described above (except for 14 specimens that were tested initially negative), and (iii) a volume of sample of at least 1.6 ml. The study was conducted on the residual clinical specimens stored at −80°C; for this reason, no informed consent was required from the patients. However, the study was submitted to the approval of the local Ethic Committee of the University Hospital of Saint-Etienne. The results of these screening tests were taken into consideration for the selection of a representative diversity of respiratory pathogens but not for the final analysis because many specimens were found positive for additional pathogens with reference to this preliminary screening.

The samples were fractionated blindly (by M.L.) into 7 tubes containing 200 µl of specimen and frozen at −80°C. For each technique tested, one aliquot of each specimen was thawed and nucleic acids were extracted using the NUCLISENS® easyMAG™ (bioMérieux) in presence of 20 µl of proteinase K (10 mg/ml) and of internal control when available, as recommended by the manufacturers (Figure 1). The nucleic acids were eluted in a volume of 100 µl for kit A, 50 µl for kits B, C, D and F, and 55 µl for kit E; for the reference method, 400 µl of sample were eluted in 100 µl. The reverse transcription and PCR reactions were performed immediately after extraction. The extracts were then conserved at −80°C for further investigation if necessary.

### Validation and Analysis of Results

When a signal was detected, it was considered as positive. The interpretation of the results obtained by each kit was validated with the acknowledgement of the corresponding supplier.

The respective sensitivity, specificity, accuracy and agreement (as evaluated by the Cohen kappa coefficient) of each tested kit were calculated by reference to the performances of the Argene/bioMérieux duplex tests used as gold standard.

## Results

### Characterization of the Panel and Overall Results

Following testing of the 88 samples with the reference method, the number of each pathogen considered to be present in the panel was as depicted in Table 2. Most pathogens, with the exception of influenza A virus, were frequently associated with at least another pathogen. The performances of each kit are summarized in Table 3 for viruses and in Table 4 for bacteria.

**Table 2:** List of pathogens that were considered to be present in the panel of the 88 specimens according the combination of duplex PCR tests (Argene/bioMérieux) used as gold standard.

Pathogens	Single infections	Co-infections		Total
		with another pathogen	with at least 2 other pathogens	
Influenza A viruses	4	3	0	7
Influenza B viruses	0	5	0	5
Respiratory syncytial virus	3	4	6	13
Metapneumovirus	0	2	2	4
Parainfluenza viruses^a^	3	4	3	10
Rhinoviruses/enteroviruses	8	5	10	23
Coronaviruses^b^	7	5	13	25
Adenoviruses	2	9	11	22
Bocaviruses	1	4	7	12
*Mycoplasma pneumoniae*	2	4	4	10
*Chlamydophila pneumoniae*	1	1	2	4
*Bordetella pertussis*	2	4	3	9
None	–	–	–	11

a type 1 (n = 2), type 3 (n = 3) and type 4 (n = 5).

b type NL63 (n = 6), type OC43 (n = 8), type 229E (n = 1), type HKU1 (n = 6) and untyped (n = 4).

**Table 3:** Performances of the 6 kits evaluated in this study for a panel of 9 viruses or groups of viruses with reference to the combination of duplex PCR tests (Argene/bioMérieux) used as gold standard.

Pathogens tested	No. of positive specimens with the reference test	Performances	Evaluated kits					
			A	B	C	D	E	F
Influenza A	7	sensitivity %	100	57.14	100	100	100	57.14
		specificity %	100	100.00	98.78	98.78	100	100
		accuracy	1.00	0.97	0.99	0.99	1.00	0.97
		kappa coefficient [95% confidence interval]	1 [1;1]	0.71 [0.40; 1]	0.93 [0.79; 1]	0.93 [0.79; 1]	1 [1;1]	0.71[0.40; 1]
Influenza B	5	sensitivity %	80.00	60.00	100	100	60.00	60.00
		specificity %	100	98.81	98.81	97.65	100	98.81
		accuracy	0.99	0.97	0.99	0.98	0.98	0.97
		kappa coefficient [95% confidence interval]	0.88 [0.66; 1]	0.65 [0.28; 1]	0.90 [0.72; 1]	0.82 [0.58; 1]	0.74 [0.39; 1]	0.65 [0.28; 1]
RSV	13	sensitivity %	92.31	92.31	100	100	92.31	69.23
		specificity %	98.68	96.15	91.46	96.15	100	100
		accuracy	0.98	0.95	0.92	0.97	0.99	0.95
		kappa coefficient [95% confidence interval]	0.91 [0.79; 1]	0.830 [0.67; 0.99]	0.74 [0.56; 0.92]	0.88[0.74; 1]	0.95[0.86; 1]	0.79 [0.60; 0.99]
Meta pneumovirus	4	sensitivity %	100	75.00	100	100	100	75.00
		specificity %	98.82	93.33	98.82	98.82	98.82	98.82
		accuracy	0.99	0.92	0.99	0.99	0.99	0.98
		kappa coefficient [95% confidence interval]	0.88[0.66; 1]	0.42[0.08; 0.77]	0.88[0.66; 1]	0.88[0.66; 1]	0.88 [0.66; 1]	0.74 [0.39; 1]
Parainfluenza viruses	10	sensitivity %	90.00	80.00	100	100	100	70.00
		specificity %	97.50	97.50	92.86	97.50	97.50	100
		accuracy	0.97	0.95	0.93	0.98	0.98	0.97
		kappa coefficient [95% confidence interval]	0.83[0.66; 1]	0.77[0.56; 0.99]	0.73[0.53; 0.93]	0.90[0.76; 1]	0.90[0.76; 1]	0.81[0.59; 1]
Rhinoviruses/enteroviruses	23	sensitivity %	86.96	39.13	91.30	82.61	100	82.61
		specificity %	91.55	100	84.42	90.28	83.33	94.20
		accuracy	0.90	0.84	0.84	0.88	0.85	0.91
		kappa coefficient[95% confidence interval]	0.75[0.59; 0.90]	0.49[0.28; 0.70]	0.64[0.50; 0.78]	0.69 [0.52; 0.86]	0.68[0.52; 0.83]	0.77[0.61; 0.92]
Coronaviruses	25	sensitivity %	80.00	24.00 a	84.00	4.00 b	76.00	48.00
		specificity %	98.44	100	98.44	100	96.92	100
		accuracy	0.93	–	0.94	–	0.91	0.85
		kappa coefficient[95% confidence interval]	0.82[0.69; 0.96]	–	0.85 [0.75; 0.96]	–	0.77[0.61; 0.92]	0.57 [0.37; 0.76]
Adenoviruses	22	sensitivity %	63.64	45.45	86.36	59.09	45.45	31.82
		specificity %	100	100	92.96	100	100	100
		accuracy	0.91	0.86	0.91	0.90	0.86	0.83
		kappa coefficient[95% confidence interval]	0.72 [0.55; 0.90]	0.56 [0.35; 0.77]	0.77 [0.61; 0.92]	0.68 [0.50; 0.87]	0.56 [0.36; 0.77]	0.41 [0.19; 0.63]
Bocavirus	12	sensitivity %	50.00	25.00	75.00	58.33	33.33	16.67
		specificity %	100	100	97.44	98.70	100	100
		accuracy	0.93	0.90	0.94	0.93	0.91	0.89
		kappa coefficient[95% confidence interval]	0.63 [0.37; 0.90]	0.37 [0.06; 0.67]	0.75 [0.54; 0.96]	0.66 [0.42; 0.91]	0.46[0.16; 0.76]	0.26[0; 0.55]
Overall(except coronaviruses)^c^	96	sensitivity %	79.17	54.17	91.67	81.25	76.04	56.25
		specificity %	98.41	98.16	94.32	97.28	97.46	99.08
		accuracy	0.96	0.92	0.94	0.95	0.94	0.93
		kappa coefficient[95% confidence interval]	0.81[0.75; 0.88]	0.61[0.51; 0.70]	0.77[0.70; 0.83]	0.79[0.72; 0.86]	0.76[0.69; 0.83]	0.66[0.58; 0.73]

a Kit having no probe for the detection of HKU1 coronavirus.

b Kit having a probe for detecting 229E coronavirus only.

c All the tested kits did not detect all the coronavirus types (see notes ^a^ and ^b^).

**Table 4:** Performances of the 2 kits evaluated in this study for a panel of 3 atypical bacteria with reference to the combination of duplex PCR tests (Argene/bioMérieux) used as gold standard.

Pathogens tested	No. of positive specimenswith the reference test	Performances	Evaluated kits	
			A	B
*Mycoplasma pneumoniae*	10	sensitivity %	70.00	80.00
		specificity %	100	98.73
		accuracy	0.97	0.97
		kappa coefficient[.95% confidence interval]	0.81 [0.59; 1]	0.82 [0.63; 1]
*Chlamydophila pneumoniae*	4	sensitivity %	50.00	50.00
		specificity %	100	100
		accuracy	0.98	0.98
		kappa coefficient[95% confidence interval]	0.66 [0.21; 1]	0.66 [0.21; 1]
*Bordetella pertussis*	9	sensitivity %	11.11	11.11
		specificity %	100	98.75
		accuracy	0.91	0.90
		kappa coefficient[95% confidence interval]	0.18 [0; 0.50]	0.15 [0; 0.45]
Overall	23	sensitivity %	43.48	47.83
		specificity %	100	99.21
		accuracy	0.95	0.95
		kappa coefficient[95% confidence interval]	0.58 [0.38; 0.79]	0.59 [0.39; 0.78]

### Sensitivity of the Tested Kits for Detecting Respiratory Pathogens

The overall sensitivity ranged from 56.25% to 91.67% for viruses (Table 3). Kits B and F exhibited the poorest sensitivity for most viral agents. The other tests were found sensitive for influenza viruses (except for kit E with influenza B virus), RSV, metapneumovirus and parainfluenza viruses, but less sensitive than the gold standard for rhinoviruses/enteroviruses, adenoviruses and bocavirus. The panel composition for coronaviruses (Table 2) explains the low sensitivity of kits B and D that detect only some types of these pathogens (Table 1). Regarding bacteria, the sensitivity of kits A and B was satisfying for *Mycoplasma pneumoniae* but of 50% for *Chlamydophila pneumoniae* and close to 10% for *Bordetella pertussis* (Table 4).

### Other Performances of the Tested Kits

The overall specificity of all the kits was excellent (>94% for all pathogens); the lowest specificity was observed for rhinoviruses/enteroviruses with kits C and E (84.4% and 83.3%, respectively). Globally, the accuracy was adequate, even if lower values were observed for rhinoviruses/enteroviruses and adenoviruses. The Cohen kappa coefficient, which reflects the agreement with the gold standard, was globally strong for viruses (>0.60) (Table 3) and moderate for bacteria (0.41–0.60) (Table 4).

### Practicability

The criteria evaluated for the technical and workflow characteristics of each kit are depicted in Table 5. Ten to 22 samples were analyzed in one run. The extraction step was controlled by the addition of an internal control in 3 of the 7 kits. Ten to 70 µl of extract were needed for analysis. After the extraction step, the hands-on time varied from 50 min to 2h15 depending on the number of reaction tube opening, the availability of ready-to-use reagents and the number of mastermix. The signal interpretation was driven by using dedicated software in only three kits. The run duration varied from 2h30 to 9 h.

**Table 5:** Practicability of each kit using the NUCLISENS easyMAG as extraction instrument (see text for correspondence between letters and commercial denominations).

	Gold standard	A	B	C	D	E	F
Throughput (number of tested specimen/run)	10	22	13	22	22	22	22
Hands on time	50 min	2h15	2h15	1h45	2 h	1h30	1h30
Run duration	2h30	7 h	7 h	5h30	9 h	4h30	4h30
Number of reaction tubes opening	0	2	1	2	1	1	3
Ready to use reagents	yes	no	no	no	yes	no	no
Addition of internal control at the extraction step	no	yes	no	no	no	yes	yes
Amplification of a cellular gene control	yes	no	yes	yes	no	no	no
Volume of extracted sample	100 µl	10 µl	33 µl	11 µl	10 µl	10 µl	10 µl
Number of mixtures	7	2	4	3	2 then 1	1	1
Software for results interpretation	no	no	yes	no	yes	yes	no
Access to raw data	yes	yes	yes	yes	yes	yes	yes

## Discussion

To our knowledge, this study is the first that compared 6 commercialized multiplex PCR techniques for the detection of respiratory pathogens. Five of these kits (A, B, D, E and F) had been previously evaluated versus conventional techniques [27]–[31], home-brew PCR [29], [31]–[35], or other commercial multiplex kits [27]–[31], [33]–[34], [36]–[41]. However, none of these studies compared more than 4 commercialized techniques simultaneously. It is noteworthy that the 6 kits tested in this study do not constitute an exhaustive panel of the techniques presently commercialized on the world market but are representative of those available in Europe at the beginning of 2011.

The present comparison includes two multiplex techniques (A and B) that were able to detect respiratory viruses together with atypical bacteria. The molecular diagnosis of these bacteria is most often performed by using home-brew methods and few commercial techniques have been evaluated comparatively [20]–[23]. The simultaneous detection of atypical bacteria and viruses represents a great advantage in terms of clinical management. Indeed, in case of positive result for an atypical bacterium, a rapid treatment with an adapted antimicrobial drug can be proposed [22]; by contrast, if this information is lacking, there is a risk for prescribing no treatment, especially if the specimen is also found positive for one or several viruses, a rather common situation with the present panel. In the opposite case, the absence of atypical bacteria (together with the negativity of conventional bacterial cultures) would allow to spare useless antibiotic treatment and consequently the emergence of bacterial resistance [7].

This study exhibits a few limitations. No gold standard is available for each of the 12 pathogens tested in this study. Even if molecular techniques are globally considered as the most sensitive, it is difficult to identify a precise method as the reference for a given pathogen or family of pathogens. In addition, the sensitivity may vary when the test is adapted to the whole family (adenoviruses, rhinoviruses/enteroviruses, parainfluenza viruses, coronaviruses…) or specific for a single type. Despite this difficulty, the Argene/bioMérieux strategy, which consists in the combination of duplex real time PCR methods, was taken as gold standard in the present study; actually, the reduced primer competition due to a combination of biplex amplification together with a higher volume of extracted sample are indicative of a good sensitivity. The low number of strains for some pathogens (i.e. *C.pneumophila*) and the absence of others (notably parainfluenza virus type 2 and *L.pneumophila*) constitute an additional limit of the study for accurate comparisons. Another difficulty for comparing these different kits relies on the level of differentiation of the tested pathogens that varied greatly from a manufacturer to another; for instance, some kits were able to detect coronaviruses and parainfluenza viruses at the type or species level whereas others considered the genus level only. For adenoviruses and rhino/enteroviruses, most of the kits used genus-specific targets. Of note, none of the kits was able to detect *Parechovirus* despite the recent involvement of this genus of *Picornaviridae* in respiratory infection [42]–[43].

In terms of sensitivity, kits B and F exhibited the lowest results, in accordance to previous evaluations in comparison to other molecular tests [28], [30], [32]–[36] despite their good performance with reference to conventional techniques [34], [44]–[46]. These “pioneer” technologies were either stopped or improved through new generation kits, as exemplified by kit C that represents a further version of kit B and exhibits the best sensitivity for all the tested viruses. The fact that the panel included a large number of multiple infections could explain some missed results with the multiplex PCR assays tested in this study; indeed, this technology favors primers’ competition, notably for the pathogen(s) exhibiting the lower load in the specimen. This observation may explain the overall low sensitivity observed for coronaviruses, adenoviruses and bocaviruses. Concerning kit D, in accordance to previous results with home-brew PCR techniques [9], [18], it exhibited a correct sensitivity except for coronavirus, essentially because it detects only the 229E type. Kit E was extensively tested with good analytical performance [27]–[28], [30]–[31], [37]–[40]; the performances observed in this study confirm these results despite a few defects, notably for adenovirus, as reported before [30], [39]. The performances of kit A were satisfactory, in accordance with previous evaluations [29], [36], [38]–[39]; the poor results obtained with *B. pertussis* led to the change of the target used for detecting this bacterium.

Regarding the technical and workflow characteristics of the kits, it is important to favor techniques with ready-to-use reagents, little number of reaction tube opening and limited number of pipeting in order to reduce the risk of mistakes in reagent handling and of cross-contamination. In several companies, this trend was a marked feature for driving the evolution of their kit. The presence of an internal control and, if possible, of a cellular control is also recommended for checking the validity of negative results. Finally, although the results of all these techniques were available in less than 12 hours, the run duration varied significantly from a technique to another. Further studies would be needed for evaluating the consequence of this difference from a clinical point of view.

In conclusion, the present study constitutes an overview of the multiplex techniques that were available in 2011 on the European market for the diagnosis of respiratory infection. Their performances are globally satisfactory, at least for those that are still commercially-available in 2013. On the basis of the present evaluation, the spectrum of detected pathogens (with an advantage for the techniques detecting also atypical bacteria), the technology used for PCR product revelation and the laboratory organization appear as determinant features for the selection of a kit. Further studies are needed for evaluating the cost-benefit of these techniques in the clinical management of respiratory infection.
